# First Insights into the Genome Sequence of the Halophilic Archaeon *Halalkalicoccus paucihalophilus* (DSM 24557)

**DOI:** 10.1128/genomeA.00382-16

**Published:** 2016-05-19

**Authors:** Anja Poehlein, Katharina Mucek, Marieke Enders, Frederik Pankok, Rolf Daniel

**Affiliations:** a Genomic and Applied Microbiology & Göttingen Genomics Laboratory, Institute of Microbiology and Genetics, Georg-August University, Göttingen, Germany; b Members of the Applied Bioinformatics in Microbiology Course of the Microbiology and Biochemistry M.Sc./Ph.D. program, Georg-August University, Göttingen, Germany

## Abstract

*Halalkalicoccus paucihalophilus* is an extremely halophilic, Gram-negative, and nonmotile coccus-like archaeon, which was originally isolated from the Lop Nur region in the northwest of China. The genome consists of a single replicon (3.98 Mbp). *H. paucihalophilus* is able to utilize mannose, which is unique for members of this genus.

## GENOME ANNOUNCEMENT

The strictly aerobic and halophilic archaeon *Halalkalicoccus paucihalophilus* was isolated from the Lop Nur region in Xinjiang Province in Northwest China (1). It is a Gram-negative and nonmotile coccus. *H. paucihalophilus* represents a new species within the genus *Halalkalicoccus*, which exhibited 99% 16S rRNA gene sequence identity to its closest relative *Halalkalicoccus tibetensis* (1). *H. paucihalophilus* (DSM 24557) was obtained from the German Collection of Cell Cultures (DSMZ; Braunschweig, Germany). The MasterPure complete DNA purification kit (Epicentre, Madison, WI, USA) was used for DNA isolation, which was followed by generation of Illumina shotgun sequencing libraries, according to the protocol of the supplier (Illumina, San Diego, CA, USA). A MiSeq reagent kit version 3 and a MiSeq instrument were used for paired-end sequencing, as recommended by the manufacturer (Illumina). Trimming of the recovered reads was performed with Trimmomatic version 0.3.2 (2) and yielded 4,625,882 paired-end reads. SPAdes version 3.6.2 (3) was employed for assembly, which resulted in 35 contigs (>500 bp), with an average coverage of 208.58×. With Qualimap version 2, the assembly was validated and the read coverage determined (4). The draft genome of *H. paucihalophilus* contained a single chromosome, with a G+C content of 61.67%. Automatic annotation, gene prediction, and identification of rRNA and tRNA genes were carried out with Prokka (5). The analyses revealed 4,163 predicted protein-coding genes, of which 2,508 had a predicted function and 1,655 were hypothetical proteins. The genome harbored 54 tRNA genes and one rRNA operon. In contrast to other members of the genus, it is reported that *H. paucihalophilus* is able to utilize mannose and galactose (1). The genome contains a putative *gmd-1* gene that encodes GDP-mannose 4,6-dehydratase. In some eukaryotes, this enzyme converts GDP-mannose to GDP-4-dehydro-6-deoxy-d-mannose and H_2_O (6). *H. paucihalophilus* is able to use galactose as a carbon source (1). Correspondingly, a gene coding for β-galactosidase and one coding for d-gluconate–d-galactonate dehydratase (*gad3*) were present in the genome. The *gad3* gene is part of the galactose catabolism via the Entner-Doudoroff pathway (7). In addition, several putative genes for glycotransferases, such as *aglD, mshA, aglI, gtf1*, and *aglG*, were identified. Among these, an archaea-specific glycotransferase-encoding gene (*agIJ*) involved in the synthesis of glycolipids was detected (8). Furthermore, a tRNA (tRNA-*selC*) that is required for the incorporation of selenocysteine into proteins was identified. Additionally, the genes *selA* and *selD*, which encode enzymes that are also necessary for the incorporation of selenocysteine, were present in the genome (9). However, *selB*, which plays an essential role in selenocysteine incorporation, was not identified in the genome sequence. *H. paucihalophilus* might have lost this ability, as proteins that typically contain selenocysteine in archaea (10) were either not present or were replaced by cysteine-containing derivatives in this organism.

### Nucleotide sequence accession numbers. 

This whole-genome shotgun project has been deposited at DDBJ/EMBL/GenBank under the accession no. LTAZ00000000. The version described in this paper is LTAZ01000000.
